# Structure–Thermal Stability Relationships and Pyrolysis Course in Cross-Linked Citronellyl and Geranyl Methacrylate/Hexyl Acrylate Copolymers: A TG-FTIR Study

**DOI:** 10.3390/polym18121515

**Published:** 2026-06-17

**Authors:** Marta Worzakowska

**Affiliations:** Department of Polymer Chemistry, Institute of Chemical Sciences, Faculty of Chemistry, Maria Curie-Skłodowska University, Gliniana 33 street, 20-614 Lublin, Poland; marta.worzakowska@umcs.lublin.pl; Tel.: +48-81-524-22-51

**Keywords:** copolymers, citronellyl methacrylate, geranyl methacrylate, TG/FTIR/QMS, DSC

## Abstract

Studies on the relationships between structure and thermal stability, as well as on the pyrolysis behavior of cross-linked citronellyl methacrylate/hexyl acrylate and geranyl methacrylate/hexyl acrylate copolymers, are presented. These insoluble and highly chemically resistant polymeric materials exhibited glass transition temperatures (*T*_g_) ranging from −55.2 °C to 7.6 °C, depending on their composition. The thermal stability of the prepared copolymers also depended on their composition and decreased with increasing geranyl methacrylate or citronellyl methacrylate content. The highest thermal stability was achieved for copolymers containing 20 wt% methacrylate and 80 wt% hexyl acrylate. The corresponding decomposition temperatures were 167 °C for citronellyl methacrylate-based copolymers and 190 °C for geranyl methacrylate-based copolymers. The thermal decomposition of the investigated copolymers proceeded in at least three stages, involving simultaneous pyrolysis processes and chemical reactions between the decomposition products. As a result, a mixture of low-molecular-weight saturated and unsaturated volatile compounds, as well as CO, CO_2_, and H_2_O, evolved. Based on the gaseous FTIR analysis, the pyrolysis pathways of the tested copolymers and the structures of the emitted saturated and unsaturated volatile products were proposed.

## 1. Introduction

Methacrylic polymers are among the most widely used materials due to their excellent physicochemical and mechanical properties. These polymers are most commonly synthesized via UV-induced polymerization processes [1,2,3,4,5]. Methacrylates find applications in numerous industries, including paper, paint, automotive, glass, optics, architecture, textiles, fabrics, and electronics [5,6,7,8,9,10,11,12]. In dentistry, methacrylates are employed in the fabrication of dentures and dental implants, cavity fillings, restoration of damaged teeth, and orthodontic appliances such as braces and aligners [4,13,14,15,16,17,18,19]. Methacrylate-based medical adhesives are also widely used in healthcare for wound closure and bonding medical devices. Furthermore, methacrylic resins are applied in orthopedic surgery as bone cements for anchoring joint prostheses and repairing fractures, thereby providing stability and support to bones and joints [20,21,22]. Biomedical applications of methacrylates also include their use as vaccine adjuvants, carriers for drugs, antibiotics, and antioxidants, protective film coatings for dosage forms, as well as excipients and binders in pharmaceutical manufacturing [23,24]. Methacrylates are particularly attractive materials for applications involving direct contact with living organisms and tissues because of their favorable properties, including low toxicity, resistance to physicochemical conditions in the oral cavity, biophysical and electrochemical neutrality, absence of taste and odor, accurate shape reproduction, durability associated with suitable mechanical properties, ease of processing, desirable aesthetic characteristics, rapid polymerization, short bonding time to dentin, negligible polymerization shrinkage, good marginal sealing, high adhesion to tooth tissues, color stability, low abrasion, appropriate elasticity, and moderate production costs [25,26,27,28,29].

Despite their extensive applications, the widespread use of methacrylates generates large amounts of non-biodegradable waste during both manufacturing and post-consumer disposal. A portion of this waste can be reduced through energy recovery by incineration [30] or through recycling processes [31,32]. However, only a limited number of methacrylic polymers, such as poly(methyl methacrylate), polyacrylonitrile, poly(acrylic acid), and superabsorbent methacrylates used in diapers, hygienic pads, and incontinence products, can be effectively recycled and reused in the production of new materials [31,32]. Over the past three decades, numerous studies have reported the synthesis of various methacrylate-based materials with tailored properties [33,34,35,36,37,38,39,40,41,42,43,44,45,46,47,48,49,50,51,52,53]. However, most methacrylate polymers developed to date are non-biodegradable or exhibit limited biodegradability, leading to their accumulation in the environment. Consequently, increasing attention has been focused on the development of more environmentally friendly polymers with properties comparable or superior to those of conventional poorly biodegradable methacrylates. Therefore, we carried out studies on the synthesis of novel meth(acrylate)-based polymers derived from meth(acrylic) derivatives of naturally occurring terpene alcohols and potato starch, as well as copolymers containing terpene-derived monomers in combination with linear, cyclic, and aromatic meth(acrylate) comonomers [54,55,56,57,58,59,60]. Encouraged by the promising properties of these materials, investigations into other copolymers containing terpene-based meth(acrylic) monomers have been continued.

The present study reports the preparation of novel methacrylic–acrylic copolymers synthesized via UV-induced polymerization of citronellyl methacrylate or geranyl methacrylate with hexyl acrylate, a commonly used linear acrylate monomer. According to the literature, hexyl acrylate is a versatile compound employed in numerous industrial applications, including the formulation of adhesives and sealants, plastic additives, textile-processing chemicals used in dyeing and finishing processes, as well as in the production of high-performance coatings, emulsion polymers, surfactants, and composite materials [61,62,63,64]. The structure of the obtained copolymers and the degree of conversion of carbon–carbon double bonds were confirmed and evaluated using FTIR and ^13^C CP/MAS NMR spectroscopy. The synthesized cross-linked citronellyl methacrylate/hexyl acrylate and geranyl methacrylate/hexyl acrylate copolymers were further investigated in terms of solvent and chemical resistance, glass transition temperature, thermal stability, and pyrolysis behavior. Based on the obtained results, a decomposition mechanism for these novel polymeric materials was proposed.

## 2. Materials and Methods

### 2.1. Materials

Citronellyl methacrylate and geranyl methacrylate were synthesized via the acylation reaction of terpene alcohols (citronellol or geraniol) with methacryloyl chloride in the presence of trimethylamine. The detailed methodology of their synthesis and purification has been described in Refs. [57,58,59,60]. The linear aliphatic acrylic monomer, hexyl acrylate, was purchased from Sigma-Aldrich, St. Louis, MO, USA. The UV photoinitiator, 2,2-dimethoxy-1,2 –diphenyl ethan-1-one, as well as the following solvents, methanol, butanol, acetone, acetic acid, ethyl acetate, tetrahydrofuran, diethyl ether, dioxane, chloroform, carbon tetrachloride, hexane, cyclohexane, and toluene, were supplied by Merck, Darmstadt, Germany. Sodium hydroxide, hydrochloric acid, and buffer solutions (pH 4, 7, and 10) were obtained from Avantor Performance Materials Poland S.A., Gliwice, Poland.

### 2.2. Preparation of Polymeric Materials

All investigated polymeric materials were synthesized via UV-induced polymerization. The compositions listed in Table 1 were exposed to UV irradiation using TL20W/05 SLV low-pressure mercury lamps emitting radiation in the range of 340–365 nm. Each composition was irradiated for 10 min at 25 °C. The obtained samples were subsequently conditioned at 60 °C for 5 h and then at 120 °C for 1 h in order to complete the polymerization process.

### 2.3. Characterization of the Prepared Polymeric Materials

#### 2.3.1. FTIR Spectroscopy

ATR-FTIR spectroscopy was used to characterize the structure of the meth(acrylate) monomers. The ATR-FTIR spectra of the monomers were recorded in the range of 600–4000 cm^−1^. The structure of the prepared polymeric materials was confirmed using FTIR spectroscopy (Tensor 27, Bruker, Karlsruhe, Germany). The FTIR spectra were collected using the KBr pellet technique in the range of 600–4000 cm^−1^ with a resolution of 4 cm^−1^ and 64 scans. The obtained FTIR spectra enabled both qualitative and quantitative analyses of the double bonds involved in the polymerization process. On this basis, the degree of conversion (*DC*) of the C=C bonds in the meth(acrylate) monomers was calculated according to the following equation:

*DC*/% = 100 × [1 − (*R*_polymer_/*R*_monomer_)]

where *R* is the ratio of the surface area of the C=C absorption band to the surface area of the C=O absorption band [65]. For the calculation of *R*_polymer_ and *R*_monomer_, the ratios of the surface areas of the C=C stretching vibration bands at 1636 cm^−1^ (methacrylic double bonds), 1618 cm^−1^ and 1636 cm^−1^ (acrylic double bonds), and 1676 cm^−1^ (ethylenic double bonds) to the surface area of the C=O stretching vibration band at 1726 cm^−1^ were determined for the polymers and monomers, respectively.

#### 2.3.2. ^13^C CP/MAS NMR Spectroscopy

Cross-polarization magic-angle spinning ^13^C CP/MAS NMR measurements were performed using a Bruker Avance 300 MSL spectrometer (Bruker, Germany) operating at a resonance frequency of 75.5 MHz. The spectra were recorded with 2048 scans and a spinning rate of 7300 Hz.

#### 2.3.3. Solubility of the Prepared Materials

The solubility of the prepared copolymers was evaluated gravimetrically at 25 °C. Water, methanol, butanol, acetone, acetic acid, ethyl acetate, tetrahydrofuran, diethyl ether, dioxane, chloroform, carbon tetrachloride, hexane, cyclohexane, and toluene were used as solvents. Polymeric samples (0.2 g) were immersed in the respective solvents for 6 months. At specified time intervals, the samples were removed from the solvents, dried, and weighed. The solubility, expressed as the percentage mass change (Δ*m*_S_), was calculated according to the following equation:

Δ*m_S_* = (*m*_1_ − *m*_2_)/*m*_1_ × 100%

where *m*_1_ is the mass of the sample before the solubility test, *m*_2_ is the mass after the test, and Δ*m*_S_ is the percentage mass change.

#### 2.3.4. Chemical Stability of the Prepared Materials

Polymeric samples (0.2 g) were immersed in selected chemical environments and stored for 6 months at 25 °C. The following media were used: 1 M NaOH, buffer solutions with pH values of 4, 7, and 10, and 1 M HCl. At specified time intervals, the samples were removed from the solutions, carefully washed with distilled water, dried, and weighed. The chemical stability, expressed as the percentage mass change (Δ*m*_R_), was calculated according to the following equation [66]:

Δ*m*_R_ = (*m*_1_ − *m*_2_)/*m*_1_ × 100%

where *m*_1_ is the mass of the sample before the chemical stability test, *m*_2_ is the mass after the test, and Δ*m*_R_ is the percentage mass change.

#### 2.3.5. Glass Transition Temperature (T_g_) of the Prepared Materials

Differential scanning calorimetry (DSC) was performed using a DSC 204 Jupiter instrument (Netzsch, Selb, Germany) to determine the glass transition temperature (*T*_g_) of the prepared polymeric materials. Samples with a mass of approximately 10 mg were placed in aluminum crucibles with pierced lids and heated from −120 °C to 150 °C. Argon was used as the purge gas at a flow rate of 40 mL/min. The heating rate was 10 K/min. Two DSC scans were performed for each sample, and the *T*_g_ values were determined from the second heating scan.

#### 2.3.6. Thermal Properties of the Prepared Materials

Thermogravimetric analysis (TG/DTG) was carried out using an STA 449 Jupiter F1 instrument (Netzsch, Germany) to investigate the thermal stability and decomposition behavior of the prepared polymeric materials. Samples weighing approximately 10 mg were heated in open Al_2_O_3_ crucibles from 40 °C to 550 °C. Helium (40 mL/min) and synthetic air (100 mL/min) were used as inert and oxidizing atmospheres, respectively. The heating rate was 10 K/min. Based on the obtained TG/DTG curves, the following parameters were determined: the temperature corresponding to 5% mass loss (*T*_5%_), maximum decomposition temperatures (*T*_max_), mass losses at individual decomposition stages (Δ*m*), and residual mass (*m_r_*).

#### 2.3.7. Simultaneous TG-FTIR Analysis

Simultaneous TG-FTIR analysis was performed using an STA 449 Jupiter F1 thermobalance (Netzsch, Germany) coupled online with an FTIR spectrometer (TGA 585, Bruker, Germany) in order to qualitatively identify the volatile products released during the thermal decomposition of the prepared polymeric materials. Gaseous FTIR spectra were collected under both inert (helium, 40 mL/min) and oxidizing (synthetic air, 100 mL/min) atmospheres. The FTIR spectrometer was connected online to the STA instrument through a Teflon transfer line with a diameter of 2 mm. The transfer line was heated to 200 °C to prevent condensation of volatile decomposition products. The gaseous FTIR spectra were recorded in the range of 600–4000 cm^−1^ with a resolution of 4 cm^−1^.

## 3. Results and Discussion

### 3.1. Structure of the Monomers and the Obtained Polymeric Materials

**Figure 1:**
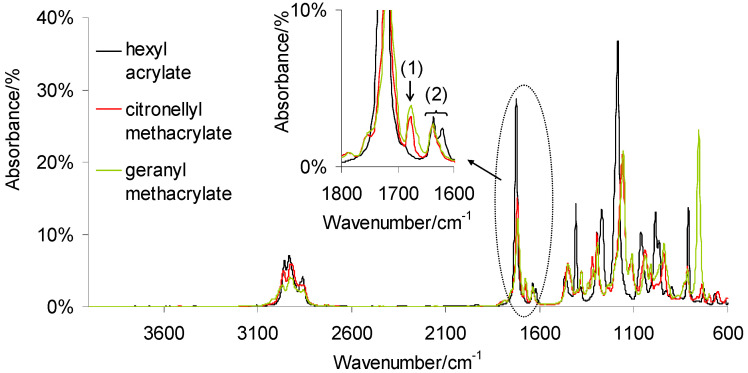
ATR-FTIR spectra for meth(acrylate) monomers.

**Figure 2:**
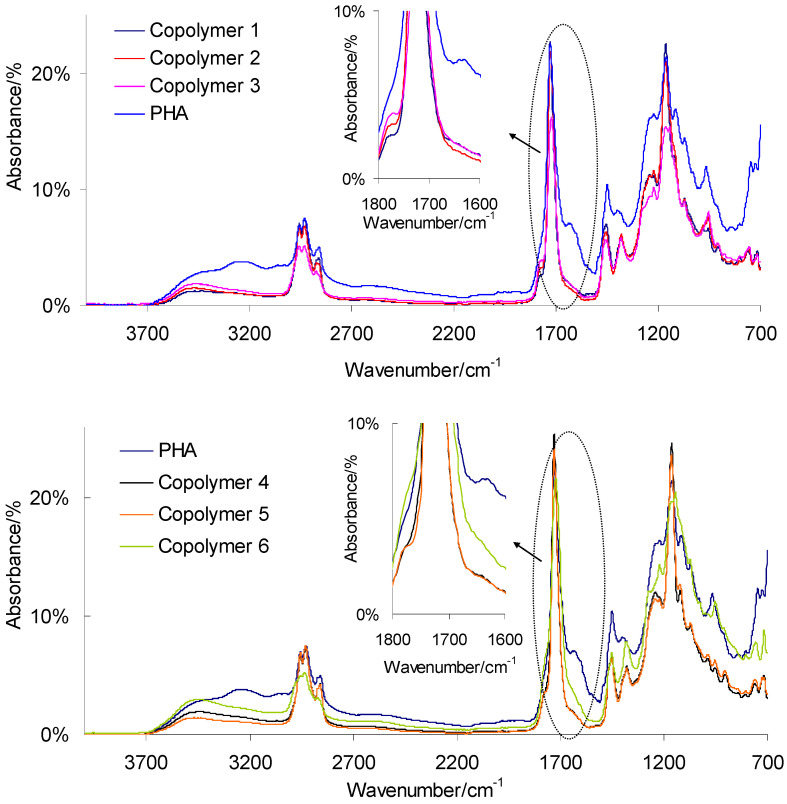
FTIR spectra of the obtained polymeric materials and poly(hexyl acrylate).

The ATR-FTIR spectra of the meth(acrylate) monomers are presented in Figure 1. The absorption bands corresponding to the stretching vibrations of the acrylic C=C double bonds, appearing as a doublet with maxima at 1618 cm^−1^ and 1636 cm^−1^, are characteristic of the hexyl acrylate monomer. In contrast, the absorption band assigned to the stretching vibrations of the methacrylic C=C double bonds at 1636 cm^−1^ is observed for citronellyl methacrylate and geranyl methacrylate. Moreover, an additional absorption band corresponding to the stretching vibrations of ethylenic C=C double bonds appears at 1676 cm^−1^ in the spectra of the methacrylate esters. A significant reduction in the intensity of the absorption bands associated with the stretching vibrations of meth(acrylic) and ethylenic C=C double bonds (bands at 1618, 1636, and 1676 cm^−1^) is observed for the UV-cured and conditioned polymeric materials [67], as shown in Figure 2. These FTIR results confirm that both types of double bonds, namely meth(acrylic) and ethylenic double bonds, participate in the polymerization process. Consequently, when mixtures of methacrylate and acrylate monomers are subjected to polymerization, polymeric materials with a cross-linked structure are formed. The calculated values of the degree of conversion (*DC*) further confirm the formation of cross-linked polymeric networks in the obtained materials (Table 2). As shown, the *DC* values exceed 90%. Such high conversion degrees clearly indicate that both types of C=C double bonds are involved in the polymerization process, leading to the formation of polymer networks with a high cross-link density [68,69].

### 3.2. ^13^C CP/MAS NMR Spectra of the Prepared Polymeric Materials

The assignments of the chemical shifts observed in the ^13^C CP/MAS NMR spectra of the investigated polymeric materials are presented in Table 3. The obtained results indicate the presence of low-intensity signals assigned to the carbonyl groups of unreacted acrylate and methacrylate moieties at 166–167 ppm. In contrast, the signals corresponding to the carbonyl groups of reacted acrylate and methacrylate units at 174–176 ppm are clearly visible [70]. The low intensity of the NMR signals associated with the carbonyl groups of unreacted acrylate and methacrylate double bonds confirms the high conversion degree of these groups during UV-induced polymerization. These results are consistent with the FTIR analysis. Moreover, the NMR spectra also confirm the participation of ethylenic double bonds originating from geranyl and citronellyl methacrylates in the copolymerization process with hexyl acrylate. Similarly to the FTIR spectra, the intensity of the NMR signals corresponding to ethylenic double bonds is low in the spectra of the investigated copolymers, which clearly indicates that these double bonds are involved in the polymerization process. As a consequence, polymeric materials with a cross-linked structure are formed.

**Scheme 1:**
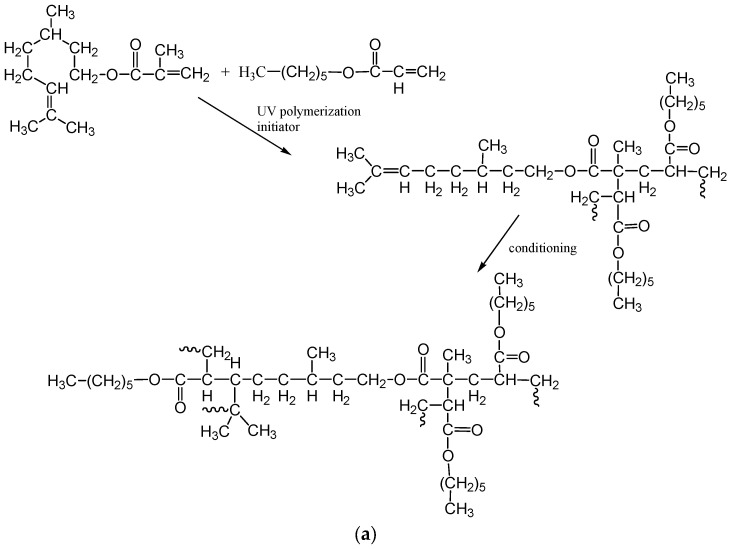

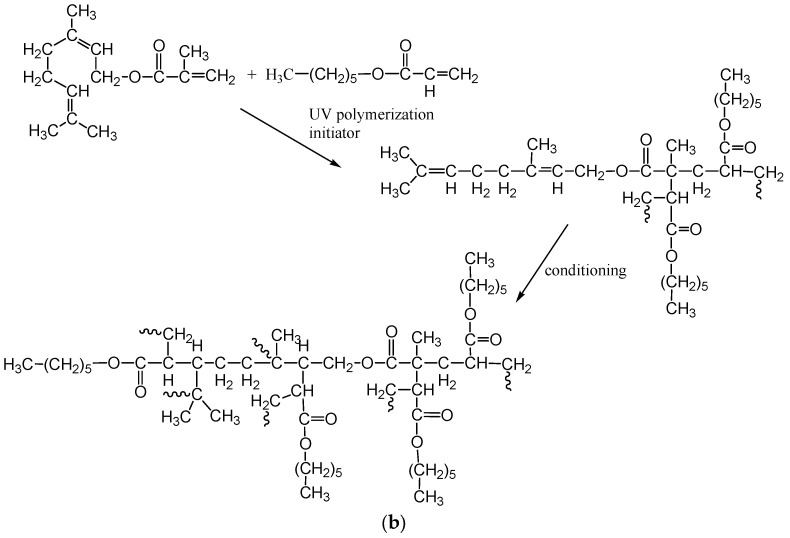
Simplified structures of the obtained (**a**) poly(citronellyl methacrylate)/poly(hexyl acrylate) and (**b**) poly(geranyl methacrylate)/poly(hexyl acrylate) copolymers.

The percentage of unreacted double bonds was also determined from the NMR spectra according to the method proposed by Rosenberg and Flodin [71] (Table 2). As can be seen, the percentage of unreacted C=C double bonds ranges from 8.3% to 11.2%, indicating a high degree of double-bond conversion, in agreement with the FTIR results. A simplified structure of the prepared copolymers, proposed on the basis of the FTIR and ^13^C CP/MAS NMR analyses, is presented in Scheme 1. As shown, the structure of the obtained polymeric materials is complex. It consists of long aliphatic chains interconnected by additional aliphatic segments forming cross-linking bridges, as well as branched aliphatic chains containing ester groups. Such a structure results in cross-linked polymeric materials characterized by a certain degree of macromolecular chain mobility due to the relatively long chain segments between individual network nodes.

### 3.3. Solubility and Chemical Resistance of the Prepared Polymeric Materials

Table 4 and Table 5 present the results of solubility tests of the prepared polymeric materials in both polar and non-polar solvents. According to these results, the prepared poly(hexyl acrylate) homopolymer is completely soluble in acetone, ethyl acetate, diethyl ether, dioxane, hexane, tetrahydrofuran, chloroform, carbon tetrachloride, and toluene. In contrast, the poly(geranyl methacrylate) and poly(citronellyl methacrylate) homopolymers are essentially insoluble in the tested solvents, which is attributed to their cross-linked structure. Their mass loss in both polar and non-polar solvents is below 1% [58,59,60]. Their practically insoluble behavior in all tested solvents confirms the formation of cross-linked polymeric networks. Moreover, the insolubility of the copolymers in solvents that readily dissolve linear poly(hexyl acrylate) confirms that copolymers free of linear homopolymer fractions were obtained. The results of chemical resistance tests are presented in Table 6. Based on these data, it can be concluded that all tested polymeric materials exhibit good chemical resistance in neutral, acidic, and basic environments, regardless of their structure. Such copolymers can be attractive materials for the production of components resistant to aggressive media, for example in the manufacture of hoses, seals, and tank linings used for transporting concentrated acids, bases, and organic solvents.

### 3.4. Glass Transition Temperature (T_g_)

**Figure 3:**
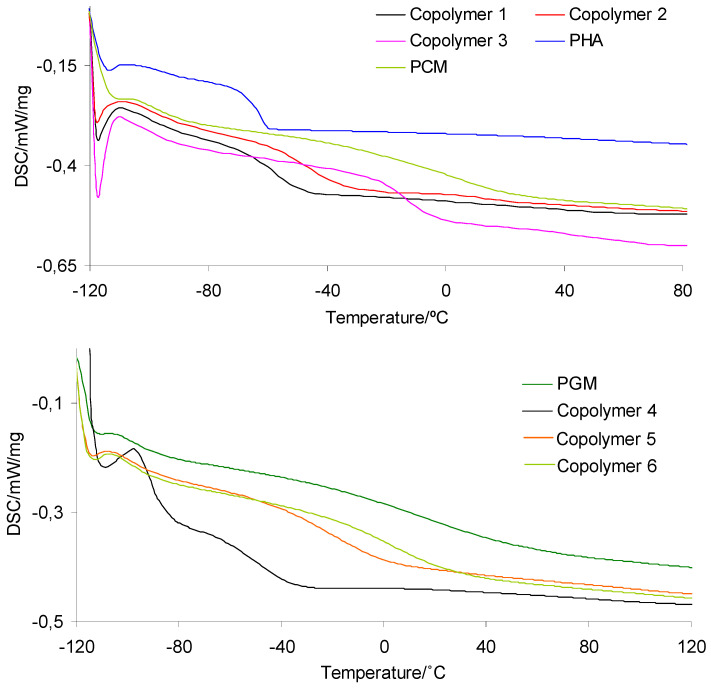
DSC curves of the tested polymeric materials.

The DSC curves are presented in Figure 3. The *T*_g_ values determined from the DSC measurements are listed in Table 7. The poly(hexyl acrylate) homopolymer is characterized by a *T*_g_ of −63.8 °C, indicating that it is a linear elastomeric material. In contrast, the poly(citronellyl methacrylate) and poly(geranyl methacrylate) homopolymers exhibit *T*_g_ values above 0 °C. Specifically, poly(citronellyl methacrylate) shows a *T*_g_ of 3.1 °C [58], while poly(geranyl methacrylate) has a *T*_g_ of 29.3 °C [60]. The *T*_g_ values of the copolymers are strongly dependent on their composition. An increase in *T*_g_ is observed with decreasing content of hexyl acrylate in the copolymer composition. In the case of citronellyl methacrylate-based copolymers, the *T*_g_ increases from −55.2 °C to −14.1 °C as the acrylate content decreases. Similarly, for geranyl methacrylate-based copolymers, the *T*_g_ increases from −44.5 °C to 7.6 °C with decreasing hexyl acrylate content. Based on the obtained *T*_g_ values, it can be concluded that most of the synthesized polymeric materials are characterized by relatively low glass transition temperatures. This indicates that the obtained amorphous copolymers consist of macromolecular chains with high mobility and flexibility at room temperature, exhibiting typical elastomeric behavior. In addition, DSC analysis shows that the prepared copolymers exhibit only a single *T*_g_, which indicates that the copolymerization proceeds between both types of monomers, leading to the formation of random copolymers [72].

### 3.5. Thermal Properties of the Prepared Polymeric Materials

**Figure 4:**
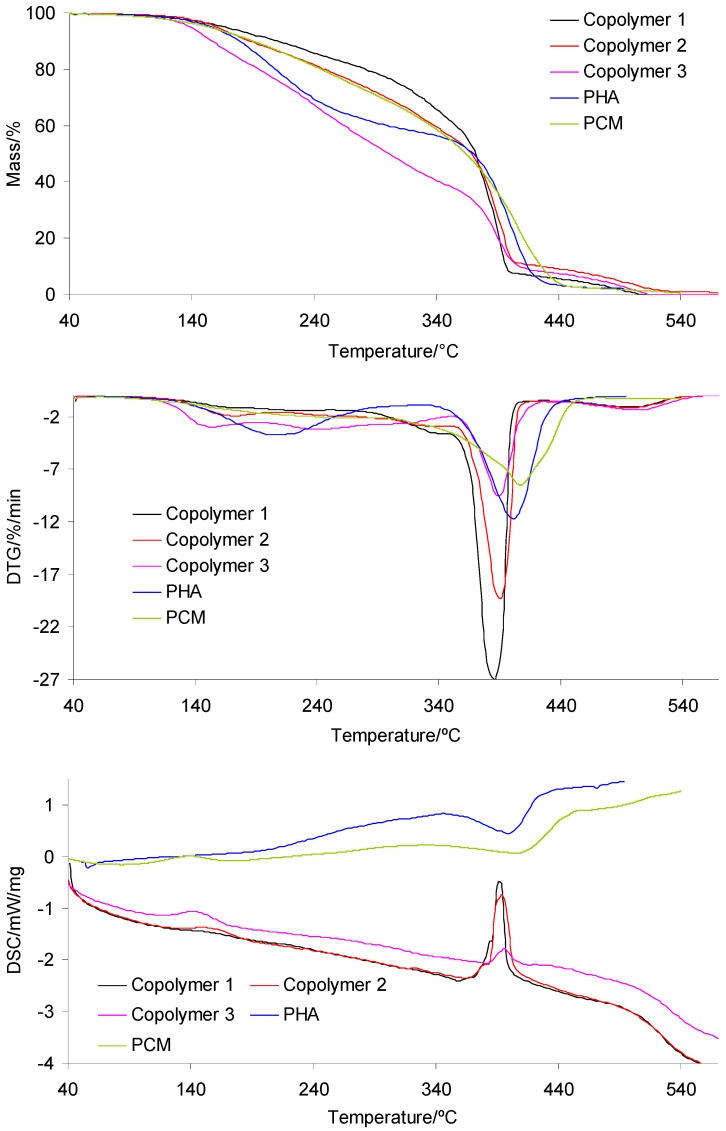
TG-DTG-DSC curves of copolymers 1–3, poly(citronellyl methacrylate), and poly(hexyl acrylate).

**Figure 5:**
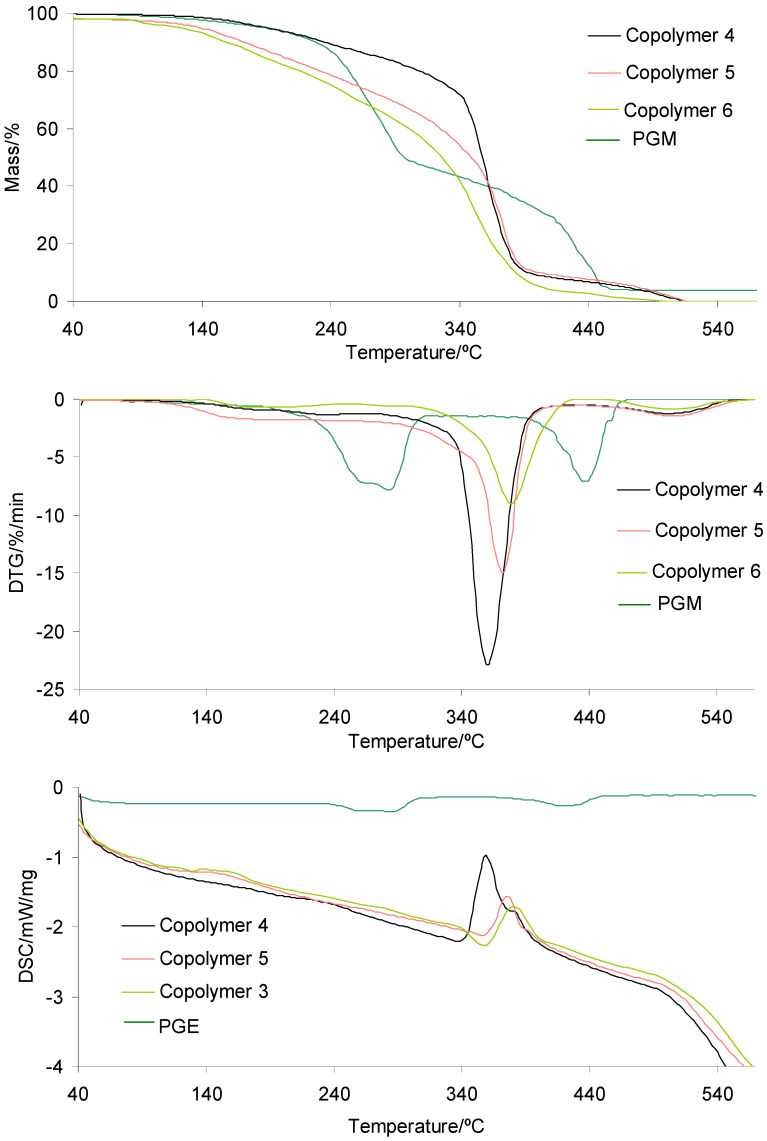
TG-DTG-DSC curves of copolymers 4–6 and poly(geranyl methacrylate).

The thermal parameters of the prepared polymeric materials are presented in Table 8 and Figure 4 and Figure 5. The TG/DTG results confirm that the thermal stability of the novel copolymers prepared from hexyl acrylate and citronellyl methacrylate (copolymers 1–3) is lower than that of the poly(citronellyl methacrylate) homopolymer [58]. On the other hand, these copolymers (1–3) exhibit higher thermal stability compared to the poly(hexyl acrylate) homopolymer. As the content of citronellyl methacrylate in the copolymer composition increases, the thermal stability decreases from 167 °C (copolymer 1) to 139 °C (copolymer 3). This behavior is somewhat unexpected and most likely results from the combined influence of both monomer units on the properties of the resulting copolymers. Similar trends are observed for copolymers 4–6 containing geranyl methacrylate, where an increase in its content leads to a decrease in the initial decomposition temperature from 190 °C (copolymer 4) to 135 °C (copolymer 6). The decrease in thermal stability with increasing citronellyl or geranyl methacrylate content may be attributed to a reduction in the activation energy of decomposition caused by the presence of these monomer units in the copolymer structure. The DTG curves indicate that all prepared copolymers undergo at least three-stage thermal decomposition under an inert atmosphere. The first decomposition stage occurs up to approximately 340 °C and appears as a low-intensity DTG signal with a maximum (*T*_max1_) in the range of 154–172 °C. The second decomposition stage, occurring from approximately 340 °C to 420 °C, is characterized by a high-intensity DTG signal with *T*_max1a_ in the range of 361–390 °C. As shown in Table 8, the total mass loss during these two stages exceeds 89% for all tested copolymers. The third decomposition stage, observed above 420 °C, is characterized by *T*_max2_ values in the range of 497–509 °C. The mass loss in this stage ranges from 2.6% to 10.6%. Very interesting results were obtained from DSC analyses performed simultaneously with TG/DTG measurements. The homopolymers prepared via UV polymerization of the respective monomers exhibit only endothermic DSC signals directly associated with bond scission and pyrolysis processes within their structures. In contrast, the DSC curves of the copolymers differ significantly. A low-intensity exothermic signal is clearly visible during the first decomposition stage. A similar behavior is observed during the second and third decomposition stages, where only exothermic signals of higher intensity are detected. These effects may result from simultaneous bond cleavage within the copolymer structure accompanied by chemical reactions between volatile decomposition products and/or reactions involving the formed residue at each decomposition stage [73,74].

### 3.6. Gaseous Decomposition Products

**Figure 6:**
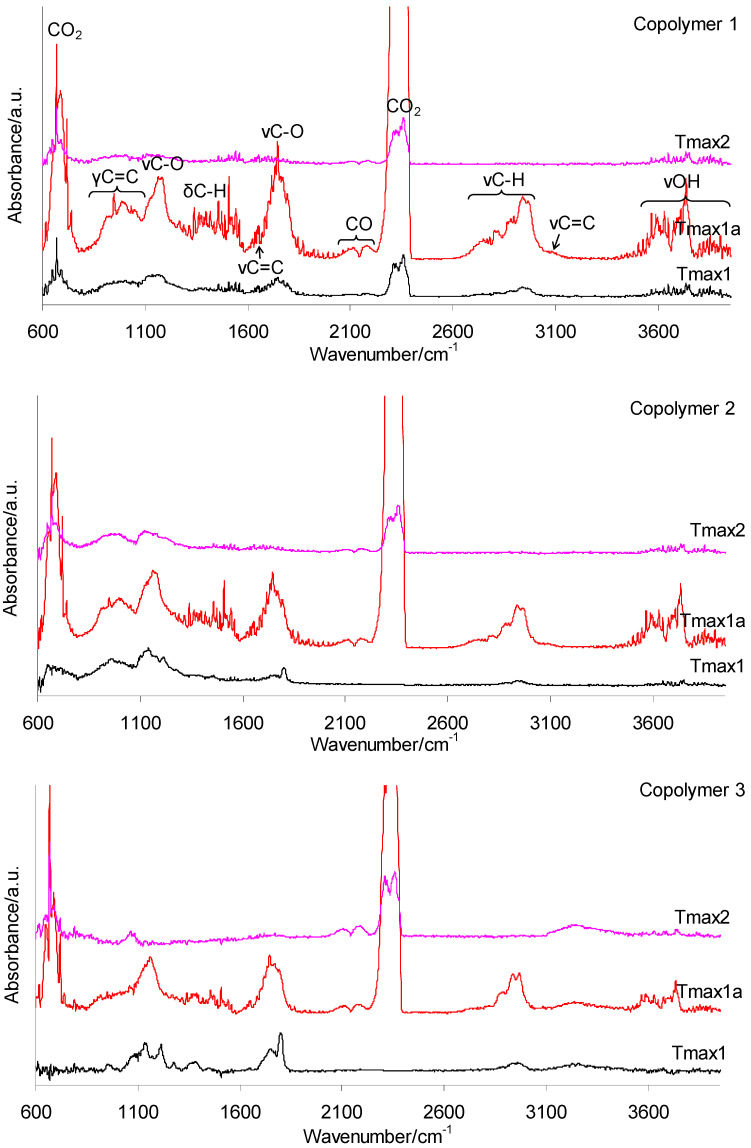

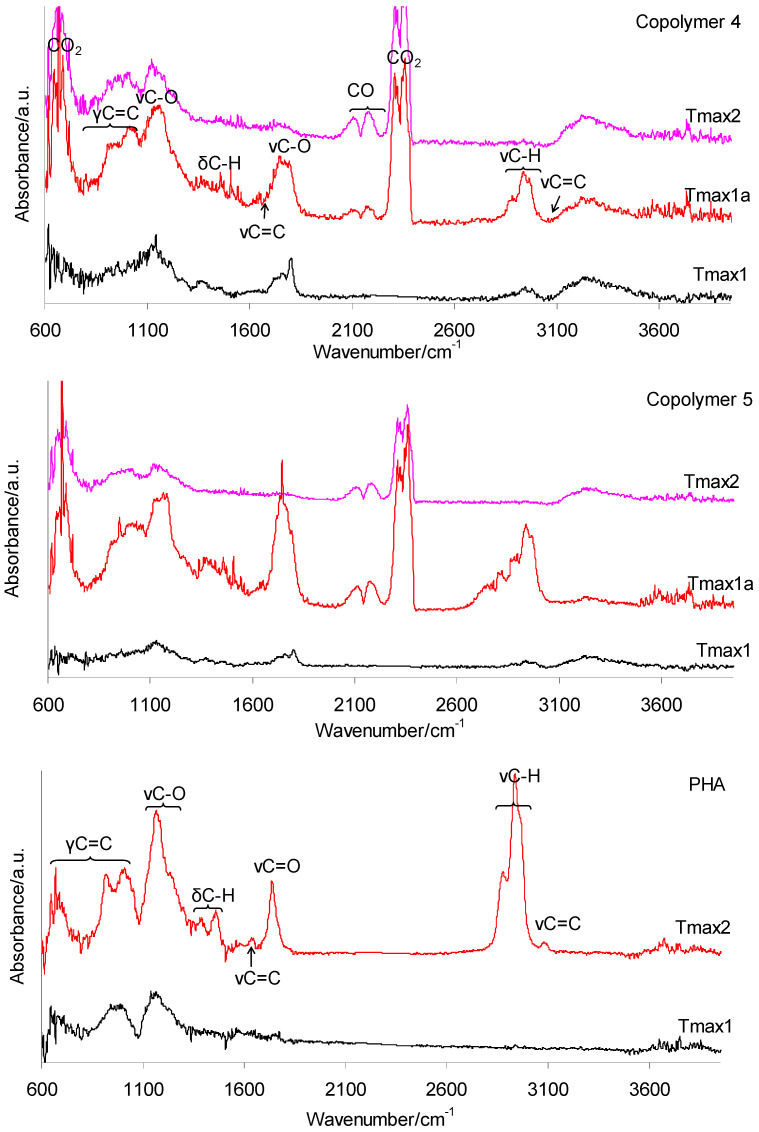

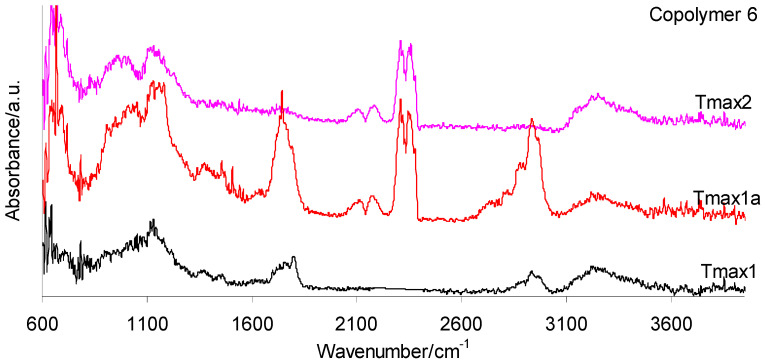
Gaseous FTIR spectra of the prepared copolymers and poly(hexyl acrylate).

**Scheme 2:**
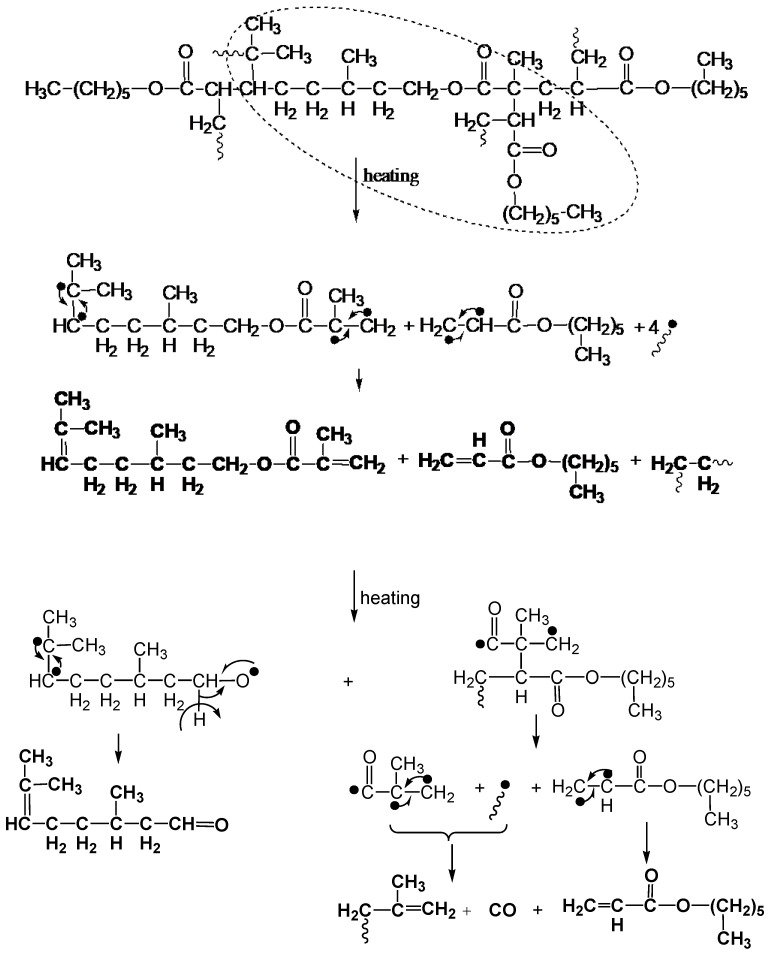

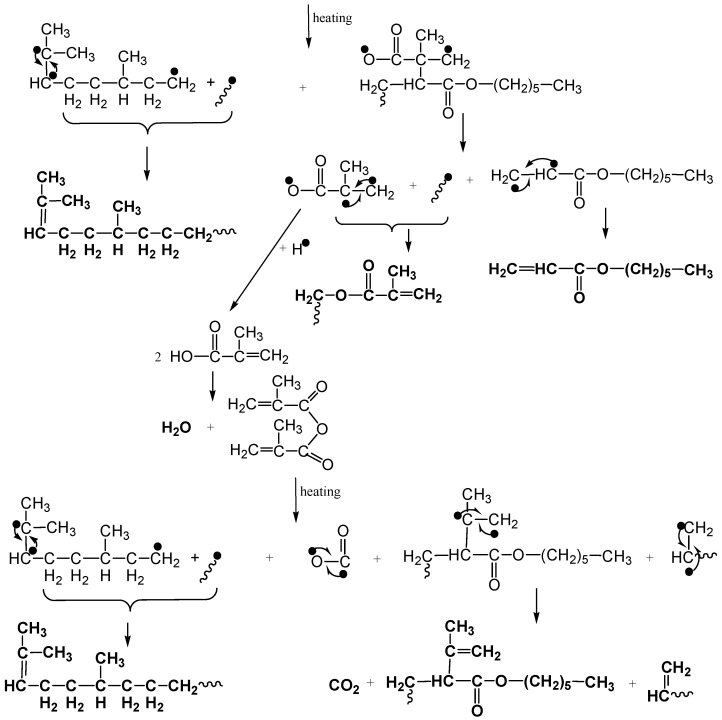
Simplified scheme of the decomposition pathway of the investigated copolymers.

Figure 6 presents the gaseous FTIR spectra recorded at the characteristic temperatures (*T*_max1_, *T*_max1a_, and *T*_max2_) for the prepared copolymers and the poly(hexyl acrylate) homopolymer (PHA). The presented FTIR spectra demonstrate that volatile products are released from the very beginning of the thermal decomposition of the investigated polymeric materials. The concentration of gaseous decomposition products at *T*_max1_ is relatively low. It increases with increasing temperature, reaching a maximum at *T*_max1a_. Interestingly, the types of emitted volatiles from the onset of decomposition up to *T*_max1a_ remain the same, while only their concentration changes. This suggests that the same types of bonds present in the structure of the investigated polymeric materials undergo pyrolysis at both *T*_max1_ and *T*_max1a_. The differences in decomposition temperatures are therefore related to different activation energies required for bond scission. It is assumed that at lower temperatures (*T*_max1_), C–C bonds located at the chain ends of macromolecules (as side branches bearing ester groups) undergo cleavage, or that macromolecules with lower molecular masses undergo depolymerization. As can be seen, at *T*_max1_ and *T*_max1a_, gases containing unsaturated C=C bonds are detected (absorption bands at 3060 cm^−1^, 1617–1646 cm^−1^, and 760–985 cm^−1^, corresponding to =C–H stretching, C=C stretching, and out-of-plane =C–H deformation vibrations, respectively). In addition, signals corresponding to –CH_3_ and –CH_2_– groups (bands at 2720–2960 cm^−1^ and 1340–1456 cm^−1^, assigned to C–H stretching and deformation vibrations), C=O groups (stretching vibrations with a maximum at 1745 cm^−1^), and C–O groups (stretching vibrations at 1114–1168 cm^−1^) are clearly observed. Furthermore, emissions of CO_2_ (bands at 2306–2354 cm^−1^), CO (bands at 2086 cm^−1^ and 2160 cm^−1^), and H_2_O (bands above 3500 cm^−1^ and in the range of 1300–1600 cm^−1^) are detected for all investigated copolymers at these temperatures. For comparison, during the thermal decomposition of poly(hexyl acrylate), volatile products containing the following functional groups are released: C=C bonds (stretching vibrations of =C–H at 3066 cm^−1^, C=C stretching vibrations at 1617–1633 cm^−1^, and out-of-plane =C–H deformation vibrations at 646–1018 cm^−1^), –CH_3_ and –CH_2_– groups (C–H stretching vibrations at 2873–2956 cm^−1^ and deformation vibrations at 1378–1448 cm^−1^), C=O groups (stretching vibrations at 1735 cm^−1^), and C–O groups (stretching vibrations at 1159–1216 cm^−1^) [67,75,76,77]. In the case of poly(hexyl acrylate) decomposition, emissions of CO_2_, CO, and H_2_O are not observed. The absence of these inorganic gases during heating of this homopolymer, as well as the lack of absorption bands characteristic of carboxyl and anhydride groups (ca. 1780–1830 cm^−1^), indicate that the homopolymer decomposes via a mechanism involving hydrogen abstraction reactions [78,79,80]. This leads to the formation of a mixture of lower-molecular-mass saturated and unsaturated ester compounds as volatile decomposition products. In contrast, the thermal decomposition of the prepared copolymers is more complex due to the presence of two different aliphatic monomer units and a more complex structure, including both main-chain bonds and cross-linking bonds between chains. Additionally, as confirmed by DSC results, reactions between intermediate gaseous products may occur during decomposition of the copolymers under an inert atmosphere. The FTIR analysis confirms the formation of volatile products containing C=C, C–H, C=O, and C–O bonds, as indicated in Figure 6. Moreover, emissions of CO_2_, CO, and H_2_O are also observed during copolymer decomposition. Taking all these observations into account, cleavage of C–C and C–O bonds in both the main chains and branched segments, together with reactions between the formed fragments, is most likely. Furthermore, the nature of the emitted volatiles suggests a radical mechanism for the decomposition of the prepared copolymers. Radical decomposition reactions proceed in an uncontrolled manner; therefore, a mixture of various degradation products is formed. The proposed general scheme based on the identified volatile products for the pyrolysis of the investigated copolymers, illustrating the course of different decomposition reactions and interactions between intermediate pyrolysis products, is shown in Scheme 2. It should be emphasized that these do not represent all possible products of radical decomposition, but only representative structures, since individual radicals can further react with each other to form a mixture of products with different molecular masses.

## 4. Conclusions

These studies confirmed that a properly designed UV polymerization process enables the preparation of polymeric materials with high resistance to solvents and chemical environments, as well as a range of glass transition temperatures depending on the composition of the individual components. UV polymerization of citronellyl methacrylate/hexyl acrylate and geranyl methacrylate/hexyl acrylate systems resulted in cross-linked copolymers with glass transition temperatures below 8 °C. All tested copolymers were thermally stable up to 135 °C, while those containing 20 wt% citronellyl methacrylate or geranyl methacrylate and 80 wt% hexyl acrylate exhibited the highest thermal stability (167 °C and 190 °C, respectively). Their pyrolysis proceeded via a radical mechanism with simultaneous reactions between the formed radical fragments. This led to the emission of a variety of unsaturated and saturated compounds (alkenes, alkanes, aldehydes, and esters with different molar masses), as well as CO, CO_2_, and H_2_O, as confirmed by TG-FTIR analysis.

In summary, the use of hexyl acrylate as a comonomer with citronellyl methacrylate and geranyl methacrylate enabled the preparation of new cross-linked polymeric materials with excellent chemical and solvent resistance, and simultaneously with different glass transition temperatures and thermal resistance compared to previously studied copolymers [58,59,60]. The incorporation of hexyl acrylate into the copolymer structure resulted in materials characterized by a low glass transition temperature together with excellent chemical and solvent resistance, rendering these elastomers promising as potential candidates for the production of various coatings or elastic components intended for contact with aggressive and organic media.

## Figures and Tables

**Table 1:** Composition of the monomer mixtures.

Polymer Name	CM/g	GM/g	HA/g	Mass Ratio of CM or GM to HA)/%
PCM	3.0	-	-	100:0
PGM	-	3.0	-	100:0
PHA	-	-	3.0	0:100
Copolymer 1	0.6	-	2.4	20:80
Copolymer 2	1.5	-	1.5	50:50
Copolymer 3	2.4	-	0.6	80:20
Copolymer 4	-	0.6	2.4	20:80
Copolymer 5	-	1.5	1.5	50:50
Copolymer 6	-	2.4	0.6	80:20

Where: CM—citronellyl methacrylate, GM—geranyl methacrylate, HA—hexyl acrylate, PCM—poly(citronellyl methacrylate), PGM—poly(geranyl methacrylate), PHA—poly(hexyl acrylate).

**Table 2:** Degree of conversion (*DC*) of double bonds determined from FTIR spectra and percentage of unreacted double bonds determined from ^13^C NMR spectra.

Polymer Name	*DC*/% (FTIR Spectra)	NMR% Unreacted C=C
PCM [58]	90	9.5
PGM [60]	92	9.2
PHA	75	26.4
Copolymer 1	90	11.2
Copolymer 2	90	11.1
Copolymer 3	93	8.3
Copolymer 4	90	11.0
Copolymer 5	91	10.1
Copolymer 6	92	10.2

**Table 3:** Assignments of chemical shifts in the solid-state ^13^C NMR spectra.

Chemical shifts/ppm	**Polymer**									
	**PCM [58]**	**PGM [60]**	**PHA**	**Cop.1**	**Cop.2**	**Cop.3**	**Cop.4**	**Cop.5**	**Cop.6**	**Group**
	175	174	175	175	176	175	174	174	175	C=O (r. C=C)
	166	165	167	167	166	167	167	167	167	C=O (ur. C=C *)
	132	131, 142	-	132	132	133	136, 142	136, 142	136, 142	=C *
	122	117	128, 130	123, 129	124, 128	123, 128	117, 130	117, 130	117, 130	=CH *
	62	63	64	63	64	63	64	64	63	-CH_2_-O-
	18–38	17–40	15–42	15–42	15–41	15–42	17–41	17–42	16–41	C–H (CH_2_, CH, CH_3_)

where: r.—reacted, ur.—unreacted, * low intensity.

**Table 4:** Percentage change of mass (Δ*m*_S_).

Polymer	Δ*m_S_/%*						
	Water	Methanol	Butanol	Acetone	Acetic Acid	Ethyl Acetate	Tetrahydrofurane
PCM [58]	0.4	0.3	0.3	0.5	0.9	0.7	0.6
PGM [60]	0.6	0.7	0.9	0.7	1.0	0.8	0.5
PHA	0.0	0.0	s	s	0.4	s	s
Copolymer 1	0.2	0.3	0.2	0.4	0.3	0.4	0.2
Copolymer 2	0.3	0.3	0.2	0.4	0.2	0.4	0.2
Copolymer 3	0.2	0.2	0.2	0.3	0.3	0.2	0.1
Copolymer 4	0.2	0.1	0.3	0.2	0.1	0.2	0.3
Copolymer 5	0.1	0.1	0.3	0.2	0.1	0.2	0.3
Copolymer 6	0.1	0.1	0.2	0.1	0.2	0.1	0.2

**Table 5:** Percentage change of mass (Δ*m*_S_).

Polymer	Δ*m_S_/%*						
	Diethyl Ether	Dioxane	Chloroform	CCl_4_	Hexane	Cyclohexane	Toluene
PCM [58]	0.3	0.9	0.6	0.6	0.8	0.6	0.7
PGM [60]	0.6	0.6	0.9	0.8	0.9	0.8	1.0
PHA	s	s	s	s	s	0.0	s
Copolymer 1	0.5	0.4	0.3	0.5	0.6	0.4	0.4
Copolymer 2	0.5	0.4	0.3	0.5	0.6	0.3	0.4
Copolymer 3	0.4	0.2	0.2	0.2	0.3	0.1	0.3
Copolymer 4	0.4	0.5	0.3	0.3	0.4	0.5	0.4
Copolymer 5	0.4	0.3	0.4	0.4	0.2	0.3	0.4
Copolymer 6	0.3	0.3	0.1	0.2	0.2	0.3	0.2

**Table 6:** Percentage change of mass loss (Δ*m*_R_).

Polymer	Δ*m_R_/%*				
	1 M NaOH	1 M HCl	Buffer (pH 4)	Buffer (pH 7)	Buffer (pH 10)
PCM [58]	0.4	0.0	0.0	0.1	0.1
PGM [60]	0.5	0.5	0.4	0.4	0.5
PHA	1.0	0.5	0.0	0.0	3.2
Copolymer 1	0.3	0.2	0.1	0.1	0.3
Copolymer 2	0.2	0.1	0.1	0.1	0.2
Copolymer 3	0.1	0.1	0.2	0.1	0.3
Copolymer 4	0.3	0.2	0.2	0.0	0.1
Copolymer 5	0.3	0.1	0.1	0.1	0.2
Copolymer 6	0.2	0.1	0.1	0.1	0.1

**Table 7:** Glass transition temperatures (*T*_g_) of the obtained polymeric materials.

Polymer	*T*_g_/°C
PCM [58]	3.1
PGM [60]	29.3
PHA	−63.8
Copolymer 1	−55.2
Copolymer 2	−41.5
Copolymer 3	−14.1
Copolymer 4	−44.5
Copolymer 5	−15.8
Copolymer 6	7.6

**Table 8:** TG/DTG data obtained under helium atmosphere.

Polymer	*T*_5%_/°C	*T*_max1_/°C	*T*_max1a/_°C	Δ*m*_1+_ Δ*m*_1a_/%	*T*_max2_/°C	Δ*m*_2_/%	*m*_r_/%
Copolymer 1	167	206/337	385	92.2	497	7.8	0
Copolymer 2	160	172/330	390	89.6	499	9.6	0.8
Copolymer 3	139	154/240	389	89.4	501	10.6	0
Copolymer 4	190	236	361	92.5	503	7.5	0
Copolymer 5	138	165	372	90.0	509	10.0	0
Copolymer 6	135	170	380	97.4	501	2.6	0
PCM [58]	251	-	396	99.2	577	0.8	0
PGM [60]	189	266/283	-	53.7	438	42.7	3.6
PHA	156	205	-	42.0	401	55.4	2.6

## Data Availability

The original contributions presented in this study are included in the article. Further inquiries can be directed to the corresponding author.
